# MDM2 and HIF1alpha expression levels in different histologic subtypes of malignant pleural mesothelioma: correlation with pathological and clinical data

**DOI:** 10.18632/oncotarget.5974

**Published:** 2015-10-30

**Authors:** Giulia Pasello, Loredana Urso, Manlio Mencoboni, Federica Grosso, Giovanni Luca Ceresoli, Francesca Lunardi, Stefania Edith Vuljan, Roberta Bertorelle, Valeria Sacchetto, Vincenzo Ciminale, Federico Rea, Adolfo Favaretto, PierFranco Conte, Fiorella Calabrese

**Affiliations:** ^1^ Department of Clinical and Experimental Oncology, Medical Oncology 2, Istituto Oncologico Veneto IRCCS Padova, Italy; ^2^ Department of Surgery, Oncology and Gastroenterology, University of Padova, Padova, Italy; ^3^ Oncology Unit, Villa Scassi Hospital, Genova, Italy; ^4^ Oncohematologic Department, Mesothelioma Unit, Oncology, SS Antonio e Biagio General Hospital, Alessandria, Italy; ^5^ Oncology, Cliniche Humanitas Gavazzeni, Bergamo, Italy; ^6^ Department of Cardio-Thoracic and Vascular Sciences, University of Padova, Padova, Italy; ^7^ Department of Clinical and Experimental Oncology, Immunology and Molecular Oncology Unit, Istituto Oncologico Veneto IRCCS, Padova, Italy

**Keywords:** mesothelioma, MDM2, HIF1alpha, inflammation, necrosis

## Abstract

Malignant pleural mesothelioma (MPM) is an aggressive tumor with poor prognosis and limited treatment options. Sarcomatoid/biphasic mesotheliomas are characterized by more aggressive behaviour and a poorer prognosis compared with the epithelioid subtype. To date prognostic and tailored therapeutic biomarkers are lacking. The present study analyzed the expression levels of MDM2 and HIF1alpha in different histologic subtypes from chemonaive MPM patients. Diagnostic biopsies of MPM patients from four Italian cancer centers were centrally collected and analyzed. MDM2 and HIF1alpha expression levels were investigated through immunohistochemistry and RT-qPCR. Pathological assessment of necrosis, inflammation and proliferation index was also performed. Molecular markers, pathological features and clinical characteristics were correlated to overall survival (OS) and progression free survival (PFS). Sixty MPM patients were included in the study (32 epithelioid and 28 non-epithelioid). Higher levels of MDM2 (*p* < 0.001), HIF1alpha (*p* = 0.013), necrosis (*p* = 0.013) and proliferation index (*p* < 0.001) were seen mainly in sarcomatoid/biphasic subtypes. Higher levels of inflammation were significantly associated with epithelioid subtype (*p* = 0.044). MDM2 expression levels were correlated with HIF1alpha levels (*p* = 0.0001), necrosis (*p* = 0.008) and proliferation index (*p* = 0.009). Univariate analysis showed a significant correlation of non-epithelioid histology (*p* = 0.04), high levels of necrosis (*p* = 0.037) and proliferation index (*p* = 0.0002) with shorter PFS. Sarcomatoid/biphasic and epithelioid mesotheliomas showed different MDM2 and HIF1alpha expression levels and were characterized by different levels of necrosis, proliferation and inflammation. Further studies are warranted to confirm a prognostic and predictive role of such markers and features.

## INTRODUCTION

**Figure 1:**
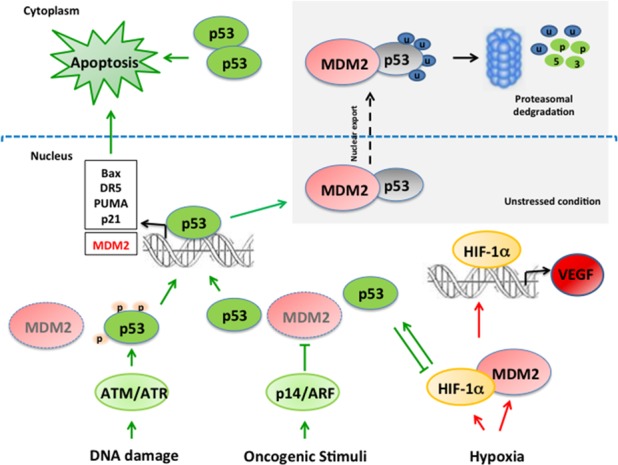
Schematic representation of MDM2/p53 and MDM2/HIF1alpha network In unstressed conditions p53 expression is kept at very low levels in the cells by the binding with MDM2 and subsequent MDM2-mediated p53 degradation. Several stress conditions such as DNA damage and oncogenic stimuli impair MDM2/p53 binding. DNA damage provokes p53 phosphorylation, preventing the interaction with MDM2 and subsequent p53 stabilization. Oncogenic stimula activate p14/ARF which inhibits MDM2 and prevents p53 degradation. MDM2 is a transcriptional target of p53 creating a negative feedback loop. Hypoxia induces increase of MDM2 and HIF1alpha, a transcription factor implied in VEGF regulation. MDM2/HIF1alpha interaction stabilizes HIF1alpha promoting its transcriptional activity. Activated p53 and HIF1alpha reciprocally modulate their activity. Indeed, HIF1alpha induces p53 activation, while p53 has a negative effect on HIF1alpha expression. MDM2: Mouse Double Minute 2; HIF1alpha: Hypoxia Inducible Factor 1 alpha; VEGF: Vascular Endothelial Growth Factor; ATM: Ataxia-Telengiectasia mutated; ATR: Ataxia Telangiectasia and Rad3-Related Protein.

Malignant pleural mesothelioma (MPM) is an aggressive tumor with increasing incidence in industrialized countries because of previous widespread exposure to asbestos and high refractoriness to chemotherapy and radiotherapy. Median overall survival (OS) and progression-free survival (PFS) with standard chemotherapy regimens are about 12 and 6 months, respectively in patients not eligible for surgery [1, 2]. Surgery is feasible in highly selected cases, and patients suitable for trimodality treatment (neoadjuvant chemotherapy, surgery, postoperative radiotherapy) achieve an overall survival that can exceed 24 months [3]. There is no standard second-line treatment for MPM, and the clinical benefits of any therapy after failure of the first line are uncertain [4–7]. Recent studies tested biologic agents targeting key oncogenic pathways, including phosphatidylinositol3-kinase (PI3K)/mammalian target of Rapamycin (mTOR) pathways, histone deacetylases (HDAC), Nuclear Factor kB (NFkB) and neoangiogenesis [8]. However, none of these therapies proved to significantly impact the natural history of this neoplasm, thus reinforcing the need for new targets and drugs in MPM. Molecular pathogenesis of MPM is characterized by several gene mutations, including neurofibromatosis 2 (NF2), BRCA1-associated protein-1 (BAP-1) and deletion of the INK4A/ARF locus (70–80%) where the genes p14/ARF and p16/INK4A are located [9]. p14/ARF is crucial in controlling cell proliferation and inhibits Murine Double Minute 2 (MDM2) protein functions [10]. MDM2 protein is normally expressed in the nucleus, while translocating to the cytosol when activated for substrates degradation, thus the significance of cytoplasmic MDM2 positive immunostaining is quite debated [11]. MDM2 is a target of p53 transcriptional function that, once activated, binds p53 to the amino-terminus for ubiquitination and subsequent proteasomal degradation [12] (Figure 1). p53 is mutated in about 50% of human cancers [13], while in tumors with wild-type p53 gene, the protein function may be lost because of MDM2 overexpression [14]. p53 reactivation through MDM2 inhibitors seems to be a promising strategy to sensitize p53 wild-type cancer cells to apoptosis, especially when MDM2 overexpression is present [15, 16]. Wild-type p53 might be present in MPM specimen [17, 18], even though few data about MDM2 expression levels are available [19].

Tumor samples of patients with MPM express high levels of neoangiogenesis markers such as HIF1alpha [20] and VEGF [21, 22], which were therefore proposed as therapeutic targets. In pathological conditions, hypoxia has been proposed as an inducer of MDM2 expression; subsequently, MDM2 could bind and stabilize HIF1alpha, responsible for VEGF transcription [23]. Moreover, inhibition of the MDM2-HIF1alpha interaction reduces VEGF mRNA expression [24] (Figure 1). These findings suggest that MDM2 might promote tumor growth through neoangiogenesis factor induction, thus representing a promising target for anticancer treatment of MPM.

Histologically, MPM is classified into three main subtypes: epithelioid (50%), sarcomatoid (16%), and mixed type or biphasic (34%). Sarcomatoid/biphasic mesotheliomas are characterized by aggressive biological behaviour, resistance to systemic treatments, more frequent distant spread and poor prognosis.

Till now molecular markers such as MDM2 and HIF1alpha together with various morphological features (proliferation, necrosis and inflammation) in different histologic types of chemonaive MPM patients have not been investigated.

According to our preliminary preclinical data, we hypothesized that MDM2 might be expressed at different levels in the two MPM histologic subtypes (Urso L. et al, 2014 International Mesothelioma Interest Group Conference).

The aim of this study was to investigate the expression levels of MDM2 and HIF1alpha in tumour samples from chemonaive MPM patients, testing different expression levels in the different histologic subtypes. Molecular markers were then correlated with different morphological and clinical data.

## RESULTS

Sixty MPM patients were included in the study: 32 epithelioid (Group 1) and 28 non-epithelioid (13 biphasic and 15 sarcomatoid) (Group 2) chemonaive mesothelioma samples. Sarcomatoid and biphasic MPM patients usually have a more aggressive disease course and a worst prognosis compared with epithelioid MPM patients. In our study population, biphasic samples were only 13 out of 60 (22%) and they showed a prevalent (higher than 50%) sarcomatoid component, thus they were grouped together in order to give more statistical power to comparisons. Median age was 70 years and there were 51 males and 9 females. Most cases were stage III, ECOG PS 1, and most patients received one chemotherapy line and no surgical treatment. Patient clinical features are summarized in Table 1.

**Table 1:** Characteristics of the study population

Patient characteristic		*N* = 60
Age		Median 70 (range 36–89)
Gender	Male Female	51 *(85%)* 9 *(15%)*
Histology	Epithelioid Biphasic Sarcomatoid	32 *(53%)* 13 *(22%)* 15 *(25%)*
Stage	I II III IV	4 *(7%)* 5 *(25%)* 26 *(43%)* 15 *(25%)*
ECOG PS	0 1 2	*17 (28%)* 36 *(60%)* 7 *(12%)*
EORTC score	Good Poor Unknown	13 *(22%)* 41 *(68%)* 6 *(10%)*
Chemotherapy lines	0 1 > 1	13 *(22%)* 34 *(56%)* 13 *(22%)*
Surgery	No EPP P/D	51 *(85%)* 2 *(3%)* 7 *(12%)*

EPP: extrapleural pneumonectomy; P/D: pleurectomy/decortication; ECOG PS: Eastern Cooperative Oncology Group Performance Status; EORTC: European Organization for Research and Treatment of Cancer.

### MDM2 and HIF1alpha expression levels in epithelioid versus non-epithelioid MPM samples

Data from MDM2 and HIF1alpha immunohistochemistry analysis were available for all the patients, while results of RT-qPCR analysis were evaluable for 46 patients, equally distributed between the two groups.

**Figure 2:**
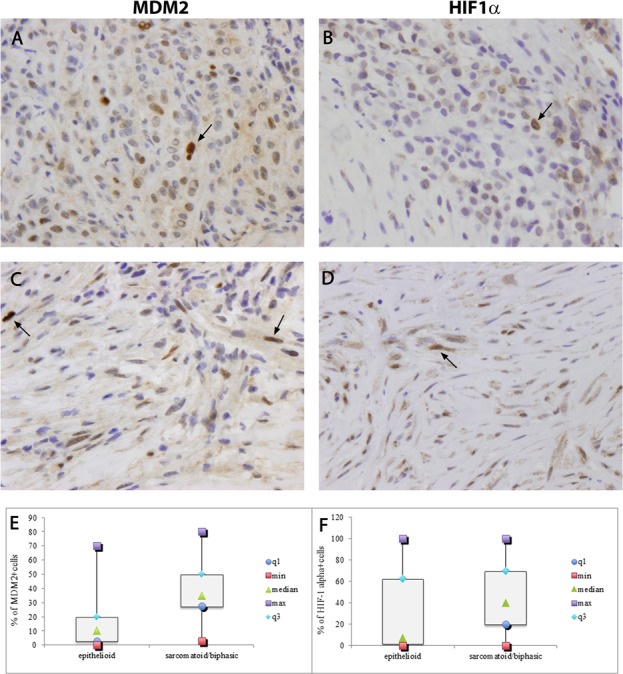
MDM2 strong nuclear immunostaining in tumor samples from epithelioid and sarcomatoid/biphasic mesothelioma. A, C. HIF1alpha strong nuclear immunostaining in tumor samples from epithelioid and sarcomatoid/biphasic mesothelioma B, D Arrays indicate emblematic strong marked nuclei. Original maginification × 320. Box plots are representative of MDM2 and HIF1alpha positive cells, and report minimum (min) and maximum (max) values, median and first (q1) and third (q3) quartile **E, F.** * statistically significant.

Higher MDM2 and HIF1alpha immunohistochemical expression levels were detected in Group 2 than Group 1 (*p* < 0.001 and *p* = 0.013, respectively) (Figure 2 and Table 2). MDM2 and overall HIF1alpha often showed cytoplasmic positive staining sometimes with dot like features, however only strongly positive nuclei of neoplastic cells were considered in the present work. Nuclear HIF1alpha was expressed by cancer cells and by intratumoral stromal cells (endothelial and fibroblasts) close to necrosis areas.

**Table 2:** Kruskal-Wallis one way analysis of variance on ranks of different levels of MDM2, HIF1alpha, inflammation, necrosis and proliferation index between the two groups

	*N*	median	25%	75%	*p* value
**MDM2 IHC**					
Group 1 (epithelioid)	32	10	3	20	
Group 2 (sarcomatoid/biphasic)	28	30	27.5	50	
Group 1 *versus* Group 2					<0.001*
**HIF-1alpha IHC**					
Group 1 (epithelioid)	32	7.5	2	62.5	
Group 2 (sarcomatoid/biphasic)	28	40	20	70	
Group 1 *versus* Group 2					0.013*
**MDM2 mRNA**					
Group 1 (epithelioid)	23	0.7	0.2	0.8	
Group 2 (sarcomatoid/biphasic)	23	0.8	0.6	1	
Group 1 *versus* Group 2					0.009*
**HIF1alpha mRNA**					
Group 1 (epithelioid)	23	0.4	0.3	0.5	
Group 2 (sarcomatoid/biphasic)	23	0.5	0.4	0.6	
Group 1 *versus* Group 2					0.173
**Inflammation**					
Group 1 (epithelioid)	32	10	5	20	
Group 2 (sarcomatoid/biphasic)	28	5	5	10	
Group 1 *versus* Group 2					0.044*
**Necrosis**					
Group 1 (epithelioid)	32	5	0	8.65	
Group 2 (sarcomatoid/biphasic)	28	5	5	16.25	
Group 1 *versus* Group 2					0.013*
**Ki67**					
Group 1 (epithelioid)	32	30	21.25	57.5	
Group 2 (sarcomatoid/biphasic)	28	65	40	70	
Group 1 *versus* Group 2					<0.001*

* statistically significant.

Higher mRNA expression levels of MDM2 were observed in Group 2 than Group 1 (*p* = 0.009) (Table 2). A moderate correlation was observed between MDM2 mRNA and protein expression levels (correlation coefficient: 0.3; *p* = 0.04).

No association was observed between HIF1alpha mRNA expression levels and histologic subtype (*p* = 0.17) (Table 2) and no correlation was seen between HIF1alpha mRNA and protein expression (correlation coefficient:−0.1; *p* = 0.5). MDM2 and HIF1alpha were not expressed in negative controls.

Interestingly, a statistically significant positive correlation was observed between immunohistochemical expression levels of MDM2 and HIF1alpha (correlation coefficient: 0.5; *p* = 0.0001). No correlation was found between mRNA expression levels of the two markers (correlation coefficient: 0.06; *p* = 0.7).

### Inflammation, necrosis and proliferation index

**Figure 3:**
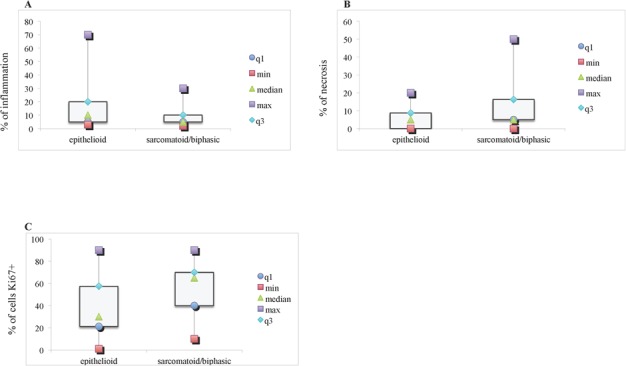
Inflammation, necrosis and Ki67 strong nuclear expression levels in tumor samples from epithelioid (*N* = 32) and sarcomatoid/biphasic (*N* = 28) mesothelioma Box plots are representative of the percentage of inflammation **A.** of necrosis **B.** within tumor samples, and of cells positive for Ki67 at the immunohistochemistry **C.** minimum (min) and maximum (max) values, median and first (q1) and third (q3) quartile are reported. * statistically significant.

Inflammation was significantly higher in Group 1 compared with Group 2 (*p* = 0.044) (Figure 3A and Table 2). Necrosis (*p* = 0.013) and proliferation index (*p* < 0.001) were significantly higher in Group 2 compared with Group 1 (Figure 3B, 3C, and Table 2).

The assessment and localization of inflammatory cells in tumor samples showed an equal distribution of inflammation in peritumoral and intratumoral areas. Interestingly, immunophenotype characterization showed a prevalent distribution of B lymphocytes (CD20+) in peritumoral areas, while T lymphocytes (CD3+) and macrophages (CD68+) were detected both at intratumoral and peritumoral levels.

Considering all patients, a significant correlation between MDM2 expression levels and necrosis (correlation coefficient: 0.34; *p*: 0.008) and Ki67 (correlation coefficient: 0.34; *p*: 0.009) was observed.

Finally, a significant correlation between necrosis and Ki67 was noted (correlation coefficient: 0.43; *p*: 0.0007).

### p53 status

p53 status was evaluated in 10 MPM samples, equally distributed between the two groups, and mutations were not detected. (Table 3). p53 protein expression was positive in all samples, with different percentage of positive cells, lower in epithelioid and higher in sarcomatoid/biphasic. p53 positive cells were 10% to 50% in epithelioid samples (median 20%), and 20% to 80% in sarcomatoid/biphasic samples (median 50%). p53 expression levels were irrespective of MDM2 higher or lower expression levels. (data not shown).

**Table 3:** p53 mutations in 10 mesothelioma samples

Patient code	Histology	MDM2 expression level	p53 mutations (exons 4–10)
P1	epithelioid	High	Wild-type
P9	epithelioid	Low	Wild-type
P11	epithelioid	High	Wild-type
P16	epithelioid	Low	Wild-type
P17	epithelioid	Low	Wild-type
P33	sarcomatoid	High	Wild-type
P37	sarcomatoid	High	Wild-type
P38	sarcomatoid	Low	Wild-type
P47	sarcomatoid	High	Wild-type
P49	sarcomatoid	Low	Wild-type

### Survival analysis

Nine patients were lost to follow-up thus no survival data were available.

**Figure 4:**
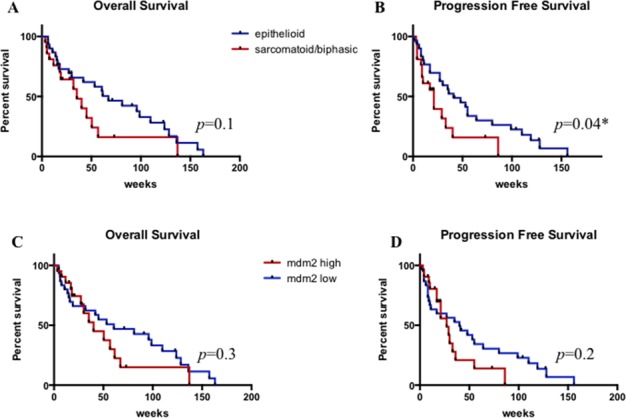
Overall survival and progression free survival according to histologic subtype. A. and B. and to MDM2 expression levels C. and D * statistically significant.

At univariate analysis, although not statistically significant, longer OS was seen in Group 1 compared with Group 2 (median OS: 67 weeks *versus* 35 weeks; *p* = 0.1) (Figure 4A). PFS was significantly longer in Group 1 than Group 2 (median PFS: 41 weeks *versus* 21 weeks; *p*: 0.04) (Figure 4B). Considering cases with low and high MDM2 expression no difference was detected in terms of OS (median OS: 60 weeks *versus* 40 weeks *p* = 0.3) or PFS (median PFS: 40 weeks *versus* 27 weeks, *p* = 0.2) (Figures 4C and 4D).

**Figure 5:**
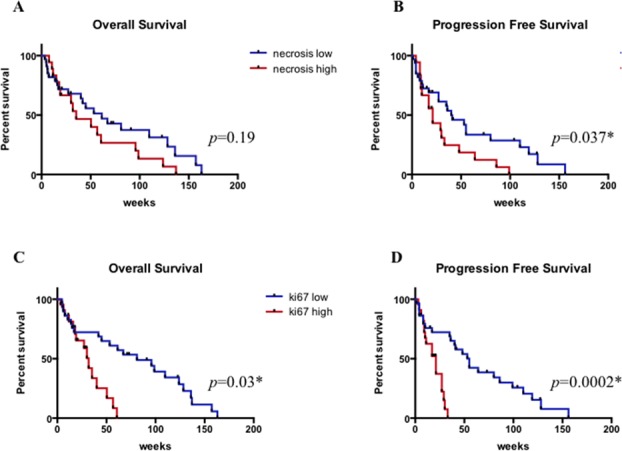
Overall survival and progression free survival according to necrosis. A. and B. and proliferation index C. and D * statistically significant.

High levels of necrosis correlated with a shorter PFS (*p* = 0.037) (Figure 5B), but not with OS (Figure 5A) and high proliferation index correlated with shorter OS (*p* = 0.03) (Figure 5C) and PFS (*p* = 0.0002) (Figure 5D).

**Figure 6:**
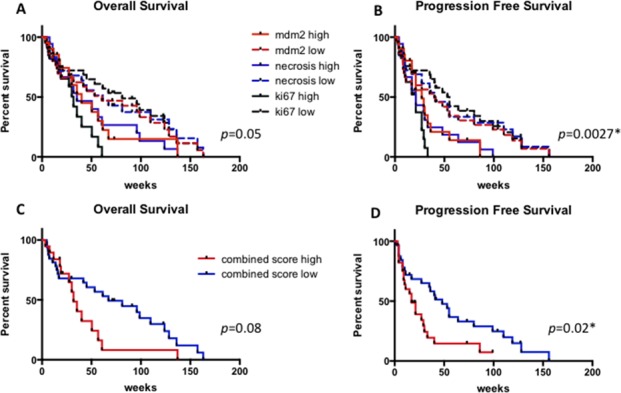
Overall survival and progression free survival in MPM patients according to high versus low levels of MDM2, Ki67 and necrosis (A, B) and according to the high versus low combined score (C and D) * statistically significant.

When considering the ‘combination score’, patients with high levels of MDM2, necrosis and proliferation index showed shorter although not significant OS (*p* = 0.08) and significantly shorter progression free survival (*p* = 0.02) (Figure 6).

Multivariate analysis showed a significant impact of EORTC prognostic score (*p* = 0.012), surgery (*p* = 0.014) and subsequent treatment lines (*p* = 0.014) on OS (Table 4). Moreover, EORTC prognostic score (*p* = 0.031) and surgery (*p* = 0.016) significantly influenced PFS, while the significant correlations observed at univariate analysis for histology, necrosis and Ki67 were not confirmed at multivariate analysis (Table 4).

**Table 4:** Covariate impact on overall survival (OS) and progression free survival (PFS) at multivariate analysis

Covariate	OS (*p* value)	PFS (*p* value)
Histology	0.47	0.49
Gender	0.97	0.27
EORTC prognostic score	0.012*	0.03*
Stage	0.29	0.17
Surgery	0.014*	0.016*
Subsequent systemic treatment lines	0.014*	NE
MDM2	0.7	0.63
HIF1alpha	0.28	0.88
Inflammation	0.31	0.84
Necrosis	0.44	0.3
Proliferation index	0.4	0.17

* statistically significant; NE: not evaluable; EORTC: European Organization for Research and Treatment of Cancer

## DISCUSSION

The poor prognosis of MPM patients, the ineffective treatment options, the absence of predictive markers for tailored treatment and the lack of knowledge about molecular pathways selectively activated in different histologic subtypes, constitute the rationale for translational studies in MPM.

Even though mutation and deletion of p53 and pRb tumour suppressor genes occur frequently in a lot of human cancers, they have been rarely described in malignant mesothelioma [18, 25].

In a previous preclinical work by our group, we showed a synergistic anticancer activity of rhApo2L/TRAIL *plus* chemotherapy, achieved through a p53-dependent upregulation of TRAIL receptors by chemotherapy, in MPM p53 wild-type cell lines and primary culture [26].

We also showed the activation of p53 by the p53-MDM2 inhibitor nutlin3a in mesothelioma cell lines, thus confirming the presence of a p53 wild type in this tumor type and the p53 reactivating properties of a MDM2-p53 inhibitor. Apoptosis assay performed in eight MPM cell lines, representing the three different histotypes (epithelioid, biphasic and sarcomatoid), showed a synergistic anticancer effect of nutlin3a *plus* rhAPO2L/TRAIL. Higher synergistic effect was shown in sarcomatoid cell lines where nutlin3a induced activation of p53 and p21, and inhibition of survivin in a dose dependent manner. Additionally, nutlin3a increased the expression of TRAIL death receptors. (Urso L. et al, 2014 International Mesothelioma Interest Group Conference). Further experiments are currently ongoing, in order to confirm a synergistic effect of the two agents *in vivo*.

These findings, the evidence of higher effectiveness of MDM2-p53 inhibitors in killing cancer cells overproducing MDM2 protein as a result of MDM2 gene amplification [27], and MDM2 disregulation in merlin-deficient tumors [28], suggest a possible role of MDM2 in malignant mesothelioma cancerogenesis and progression.

Few works have investigated the role of MDM2 expression in MPM and case series were represented mostly by epithelioid forms, thus making it difficult to draw any conclusion about different expression levels between the two main histologic subtypes [19, 29]. Importantly, it was not clear if immunohistochemical and RT-qPCR analyses were performed on chemonaive samples, and no details about systemic and locoregional treatments of affected patients were reported, which might have some impact on survival data. In line with these previous studies, we also focused our attention and further correlation studies only on the strong nuclear MDM2 expression, considering the lack of correlation between cytoplasmic protein and mRNA expression levels (data not shown) and the mild and diffuse cytoplasmic staining across tumor samples, without significant difference between the subgroups.

Our results showed that strong MDM2 overexpression- both in immunohistochemistry and in RT-qPCR- was significantly correlated with sarcomatoid/biphasic histotype. The correlation test between mRNA and protein analysis was positive, even if the positive relation was observed in only half examined tumour samples. These results might be explained in different ways. First of all, literature data confirm that MDM2 protein overexpression is not only determined by gene amplification, but also by other mechanisms, such as transcriptional and post-transcriptional modifications [30]. Secondarily, immunohistochemistry detected only strong nuclear expression, while RT-qPCR quantified mRNA derived from the whole tumor sample. Moreover, different isoforms of the protein might not reflect the overall MDM2 expression, which in turn is influenced by several factors such as altered rates of transcription, mRNA stability, enhanced translation, and diminished destruction of the protein. All will affect intracellular levels of MDM2.

MDM2 protein overexpression with or without increased gene copy number occurs more frequently in those tumors with a wild-type p53 [14]. The evidence of a p53-independent activity of MDM2 [15, 31–35] might explain the possible coexistence of both protein overexpression which seems to confer a worse prognosis to cancer patients.

Recent retrospective data of Next-Generation-Sequencing in 123 MPM tumor samples showed high frequency of mutations in the p53-DNA repair pathway, with high ratio of non synonimous-synonimous variation in TP53 and CDKNA2A suggesting a central role in MPM carcinogenesis and progression [36]. However, in line with previous literature data, we observed no p53 mutation across exons 4 through 10 in ten MPM samples (5 epithelioid and 5 sarcomatoid/biphasic), even in those samples with MDM2 overexpression.

An interesting finding of our study was the significant correlation between HIF1alpha expression levels and the sarcomatoid/biphasic histotype, as well as the correlation between expression levels of MDM2 and HIF1alpha.

To date, no literature data are available about different levels of inflammation and necrosis across mesothelioma histotypes and overall correlation between molecular markers as MDM2/HIF1alpha and morphological changes. The prognostic significance of necrosis in mesothelioma was reported by other groups, even though these results were limited to biphasic [21] or epithelioid histotype (Husain AN et al, 2014 International Mesothelioma Interest Group Conference).

Our results reported a statistically significant correlation of high proliferation index and necrosis with sarcomatoid/biphasic histology, and of high inflammation with epithelioid subtype. MDM2 expression levels significantly correlated with necrosis and proliferation index. All these findings support the concept of a different biological signature of the two cancer types. The sarcomatoid histotype could present necrosis following a failure of blood supply, as usually occurs in tumours with a great proliferative index.

Thus, from our results, it is tempting to speculate that MDM2, Ki67 and necrosis might be considered as important diagnostic parameters to characterize a more aggressive phenotype of MPM: in fact, when considering the ‘combination score’, patients with high levels of two out of three markers showed shorter OS and PFS. These results underline the importance of morphological combined with immunohistochemistry data in a tumor sample evaluation, especially when the pathologist wants to give to the medical oncologist further information about the cancer behaviour. Modern methods recently introduced in the diagnostic practice, such as tissue microarray, can't catch morphologic features such as necrosis thus darkening a face of the whole “picture”.

The prognostic significance of MDM2 overexpression is quite controversial in the literature [37]. We found a trend towards a negative prognostic and predictive significance of high MDM2 strong nuclear expression levels, thus confirming previous results in different case series [19, 29].

Sarcomatoid/biphasic histology, high levels of necrosis and proliferation index were the only features associated with shorter PFS, thus defining a more aggressive and chemoresistant phenotype. At multivariate analysis clinical features such as EORTC prognostic score, surgery and further chemotherapy after first-line treatment were the only factors significantly associated with OS and PFS. In our case series, the lack of statistically significant correlations with survival data may be caused by several factors such as the small sample size, especially for sarcomatoid/biphasic samples whose survival data were available in a small number of patients; the insufficient follow-up time of the last patients might have some impact on prognosis.

In conclusion, our study confirmed different biological and pathological features and molecular markers expression in the two main histologic subtypes of MPM. Sarcomatoid/biphasic mesothelioma is characterized by higher levels of MDM2, HIF1alpha, necrosis and proliferation index, compared with the epithelioid subtype, which in turn is characterized by higher levels of inflammation.

For the first time, our study showed a significant correlation between expression levels of MDM2 and HIF1alpha. This has relevant therapeutic implications especially for possible targeted therapies.

## MATERIALS AND METHODS

### Patient samples and data collection

We centrally collected and analyzed epithelioid, biphasic and sarcomatoid samples from the diagnostic biopsies of MPM patients who were referred to four Italian cancer centers, since 2007. Chemonaive patients were considered eligible if they had a histological diagnosis of MPM, and adequate tumor samples for immunohistochemistry and quantitative reverse transcription polymerase chain reaction (RT-qPCR). Immunohistochemistry was performed on serial sections of each paraffin block. Eight sections (10 um thickness) of the same blocks were used to perform RT-qPCR. The first patient was included in April 2007 and the last patient in November 2014.

Surviving patient follow-up was censored on March 15, 2015.

As negative controls we collected and analyzed pleural samples from patients who had undergone surgical procedures for non oncological diseases.

Clinical information about patients enrolled in the study was retrospectively collected: age, gender, Eastern Cooperative Oncology Group (ECOG) performance status (PS), European Organization for Research and Treatment of Cancer (EORTC) prognostic score, stage, systemic treatments, surgery, radiotherapy, first progression, last follow-up date, status (living/deceased).

PFS was assessed from the date of enrolment to the date of disease progression to the first-line (or relapse) or to the date of death, whichever occurred first. OS was assessed from the date of enrolment to the date of death.

To perform the statistical analyses, all data collected were recorded in a computer database with password protection.

Written informed consent was given by all the subjects included in the research. The study was approved by the Ethical Review Board of the Coordinator Center and all participating centers (CE IOV: 2013/41; approved on July 8, 2013). All procedures were in accordance with the Helsinki Declaration of 1975, as revised in 1983.

### mRNA expression analysis

Eight slices of 10 μm sections/samples (including a range of 30%–50% of neoplastic cells on entire tissue sample) were collected in 1.5 ml of a microcentrifuge tube and incubated in xylene at 50°C for 3 minutes to solubilize and remove paraffin from the tissue block, then washed two times in absolute ethanol to remove the xylene. Total RNA extraction of the deparaffinized samples was performed using RecoverAll Total Nucleic Acid Isolation Kit (Ambion) according to the manufacturer's protocol.

Although DNase treatment was included in the RNA Isolation Kit, a further DNase treatment (Invitrogen) was performed according to the manufacturer's protocol. Reverse transcription of total mRNA was performed using an equal volume (10 uL) of total RNA/sample, previously treated with DNase, by SuperScript II Reverse Trancriptase (Invitrogen) according to the manufacturer. Quantitative analysis of MDM2 and HIF1alpha genes were performed by LightCycler 480 Real time PCR System and LightCycler 480 SYBR Green I Master Mix (Roche) according to the manufacturer's protocol using specific primers for each gene (Sigma). As an internal reference we used glyceraldehyde 3-phosphate dehydrogenase (GAPDH) and β-actin. Primer pairs were designed to generate amplicons of about 50–70 base pairs in length. The sequences of the primers were as follows: MDM2 forward: 5′-GGGAGTGATCAAAAGGAC CT-3′, reverse: 5′-CCAAATGTGAAGATG AAGGTTTC-3′; HIF1alpha forward: 5′-GACAAA GTTCACCTGAGCCTAA-3′, reverse: 5′-TCATT GACCATATCACTATCCACA-3′; GAPDH forward: 5′-CAAGCTCATTTCCTGGTAT-3′, reverse 5′ATGAG GTCCACCACCCTGT-3′; β-Actin forward: 5′-AGCCTTC CTTCCTGGGCAT-3′, reverse 5′-TGGAGTTGAAGG TAGTTTCGTG-3′. Real time reaction was carried out as follows: 95°C for 5 min, 40 cycles of 95°C for 10 s, 60°C for 10 s, and 72°C for 10 s. All reactions were run in triplicate and relative quantification of gene expression was calculated using the LightCycler Relative Quantification Software (Roche) by the E (efficiency) Method [37].

### Immunohistochemistry and pathologic assessment

The presence of necrosis and inflammation was evaluated on haematoxylin and eosin stained sections and quantified using a score system from 0 to 3 (0: absent; 1: < 10%; 2: from 10 to 20% and 3 > 20% of the whole tumor section examined). Necrosis and inflammation were considered high when present in more than 5% and 9% of tumor samples, respectively.

Serial sections of 4 μm were immunostained with monoclonal antibodies for Ki67 (Clone MIB-1, Immunotech) and MDM2 (clone IF2, Life Technologies), and polyclonal antibody for HIF1alpha (Invitrogen). Only strong dark Ki67 stained MDM2 and HIF1alpha nuclei were counted and expressed as percentage of total cell number.

Cut-off values for each marker and pathological feature were set over and under the median value calculated on all the tumor samples. Ki67 positive cells percentage represent proliferation index and was considered high when above 50%. MDM2 and HIF1alpha positive cell percentage was considered high when higher than 20% and 30%, respectively. MDM2 immunohistochemistry was validated on reference tissues using liposarcoma samples.

We further stratified patients according to a combined score based on the association of MDM2, necrosis and proliferation index. The score definition was as follows: high combined score if two of three markers were high, and low combined score if two of three markers were low. Inflammatory cell characterization was performed by immunohistochemistry (IHC) in 4 μm-thick sections using the following antibodies: anti-CD45 (1:100, Biocare), anti-CD4 (1:200, Dako), anti-CD8 (1:200, Dako) and anti-CD20 (1:200, Dako). Immunoreactivity was expressed as percentage of positive cells/total inflammatory cells. Negative controls for non specific binding were processed omitting the primary antibodies and revealed no signal.

IHC for p53 was performed on the same subset of samples where p53 sequencing was available by using the monoclonal antibody anti-p53 (1:100, Cell Marque).

### p53 mutational analysis

In the COSMIC, Catalogue of Somatic Mutations in Cancer, nine mutations are reported for 147 analysed mesotheliomas, and exons 5, 7, 8, 10 were involved. The IARC, International Agency for Research on Cancer, dataset also reported 10 TP53 mutated cases in exon 5, 7 and 8. Therefore, TP53 mutations were investigated by analysing exons 4 to 10, where the majority of mutations are localized.

Briefly, DNA isolated from formalin-fixed tumor tissues was subjected to PCR using primer pairs specific for each exon by the IARC protocol. The amplified products were then sequenced by fluorescent capillary electrophoresis (ABI PRISM 310 genetic analyzer, Applied Biosystems) and sequences were compared with NCBI Reference Sequence NC_000017.10.

### Statistics

Cut-off values for definition of low *versus* high expression levels of each marker and pathological parameters were identified over and under the median value, and data are presented as box plots. The Kruskall-Wallis test was performed to evaluate a different expression of molecular markers and morphological parameters in the two histological subtypes. Correlations between MDM2 and HIF1alpha expression levels, between RNA and protein expression levels of each marker, and between MDM2 or HIF1alpha and necrosis, inflammation or Ki67 were investigated through the Spearman linear correlation analysis. OS and PFS curves were designed according to the Kaplan-Meier method for the whole patient population and for patients with a follow-up time of at least 24 weeks.

Univariate (log rank Mantel Cox test) and multivariate (Cox Regression Proportional Hazards Model) analyses were performed to show any possible impact of molecular markers, pathological parameters and clinical features on PFS and OS. Necrosis, proliferation index and MDM2 levels were also combined in a “combined score” and considered in OS and PFS analyses.
